# Anti-filarial antibodies are sensitive indicators of lymphatic filariasis transmission and enable identification of high-risk populations and hotspots

**DOI:** 10.1016/j.ijid.2024.107194

**Published:** 2024-10

**Authors:** Harriet Lawford, Helen Mayfield, Filipina Amosa-Lei Sam, Satupaitea Viali, Tito Kamu, Gretchen Cooley, Ashley Simon, Diana Martin, Colleen L Lau

**Affiliations:** 1 UQ Centre for Clinical Research, The University of Queensland, Brisbane, Queensland, Australia; 2 National University of Samoa, Apia, Samoa; 3 School of Medicine, Faculty of Health Sciences, National University of Samoa; 4 Oceania University of Medicine, Apia, Samoa; 5 Tupua Tamasese Meaole Hospital, Apia, Samoa; 6 US Centers for Disease Control and Prevention, Atlanta, GA, USA; 7 Division of Parasitic Diseases and Malaria, US Centers for Disease Control and Prevention, Atlanta, GA, USA

**Keywords:** Lymphatic filariasis, Antifilarial antibodies, Seroprevalence, Age-specific associations, Samoa

## Abstract

**Objectives:**

Circulating filarial antigen (Ag) is used by elimination programs to monitor lymphatic filariasis (LF) transmission; however, antifilarial antibodies (Ab) may be more sensitive than Ag for detecting LF. Our objectives were to describe Ab seroprevalence, identify risk factors for Ab seropositivity, investigate age-specific associations between Ag and Ab, and evaluate geographic clustering of seropositivity.

**Methods:**

Community-based serosurveys of participants aged ≥5 years were conducted in 35 primary sampling units (PSUs). Ag-positivity was detected using Alere™ Filariasis Test Strips and Ab-seropositivity using multiplex bead assays. Seroprevalence was adjusted for study design.

**Results:**

Of 3795 participants (range:5-90 years), adjusted prevalence for Ag, *Bm14* Ab, *Wb123* Ab, and *Bm33* Ab were 3.7% (n=117), 20.3% (n=583), 32.2% (n=987), and 51.0% (n=1659), respectively. Male sex, older age, and residents of suspected hotspots had higher odds of seropositivity to all seromarkers. Seroprevalence was lower in 5-9-year-olds vs ≥10-year-olds (*P<*0.001). Clustering was significantly higher in households (intra-cluster correlation for Ag:0.45; *Bm14* Ab:0.32; *Bm33* Ab:0.31; *Wb123* Ab:0.29) compared to PSUs or region.

**Conclusions:**

Abs enabled identification of risk factors for seropositivity and geographical clustering to inform targeted interventions for LF programmes. Further research is needed to define Ab thresholds for active versus past infection and elimination targets.

## Highlights

• Antibodies (Abs) were a more sensitive indicator of transmission than antigen (Ag).
• Higher seroprevalence of *Bm14* Ab*, Bm33* Ab, and *Wb123* Ab compared to Ag was found.
• Ab seroprevalence was significantly higher among older males in suspected hotspots.
• Significant clustering of Ab seropositivity was seen at the household-level.
• Further research is needed to define Ab thresholds for active versus past infection.

## Introduction

Lymphatic filariasis (LF), a mosquito-borne helminth infection caused by the filarial nematodes *Wuchereria bancrofti, Brugia malayi*, and *Brugia timori*, is an important cause of chronic disability globally. Multiple demographic and environmental risk factors for LF have been identified, which vary geographically due to the behavioural versatility and diversity of both the mosquito vectors and filarial species. In the Western Pacific Region, where filarial worms are diurnal sub-periodic [1], known risk factors include male sex [2], increasing age [2,3], lower household socioeconomic status [4], and living in a known hotspot [5].

The Global Programme to Eliminate LF (GPELF) was launched by the World Health Organization (WHO) in 2000 with the aim to (i) interrupt transmission through mass drug administration (MDA) of anthelminthic medicines, and (ii) control morbidity of affected populations by 2020 [6]. Since its start, the number of LF infections has reduced by 74% globally and as of 2022, 19/72 endemic countries have achieved elimination of LF as a public health problem [7]. New milestones and targets beyond 2020 are being developed, outlined in the *WHO Neglected Tropical Diseases Roadmap 2030*, which aims for 58/72 (81%) of countries to have eliminated LF by 2030 [8]. It further proposes that all countries complete their MDA programmes and implement post-MDA or post-validation surveillance by 2030 [9].

Despite multiple MDA rounds, several countries including Samoa have reported persistent foci of LF transmission. Resurgence has also been reported by countries that have reached very low antigen (Ag) prevalence based on Transmission Assessment Surveys (TAS) of young children [10]. Measuring antibodies (Ab) to LF Ags (including *Wb123* Ab, *Bm33* Ab, and *Bm14* Ab) may be more sensitive than Ag testing alone [11], particularly in areas with low population-level prevalence of microfilariae (Mf) and Ag, which make the identification of residual positive cases increasingly challenging [11]. Antifilarial Abs can be detected before Ag and Mf [12], providing earlier indication of infection and allowing for earlier intervention. Testing serum for LF Abs using serologic techniques such as multiplex bead assays (MBA), which measure Ab seroprevalence to multiple diseases simultaneously, also meets integrated approaches to epidemiologic surveillance of neglected tropical diseases as stressed in the *WHO Neglected Tropical Diseases Roadmap 2030* [8].

Samoa, a tropical island in the Western Pacific Region, remains endemic for LF despite continued elimination efforts since 1965 [13]. Samoa underwent 8 national MDA rounds using diethylcarbamazine and albendazole between 1999 and 2011, and 2 targeted MDA rounds in 2015 and 2017 [14]. A nationwide TAS was conducted in 2013, in which 2 of 3 evaluation units (EU) passed target thresholds, and a second TAS took place in 2017 where all 3 EUs failed [14]. In 2017, Samoa prepared a National Action Plan to Eliminate LF, and in August 2018, Samoa was the first country in the world to distribute nationwide triple-drug MDA (ivermectin, diethylcarbamazine, albendazole [IDA]) [14]. A second round of IDA MDA was planned in 2019 but was delayed due to a measles outbreak in 2019 and the global COVID-19 pandemic. It was subsequently delivered in September 2023.

The Surveillance and Monitoring to Eliminate LF and Scabies from Samoa (SaMELFS) project is an ongoing operational research program to monitor and evaluate the effectiveness of triple-drug MDA on LF transmission [14]. The SaMELFS 2018 survey took place immediately following national distribution of IDA; this study found a 3-fold higher Ag prevalence among participants *≥*10 years old compared to those 5-9 years old, significantly higher Mf prevalence among participants *≥*10 years old compared to those 5-9 years old, and significant clustering of Ag and Mf at the household level relative to the primary sampling unit (PSU) and regional level [14]. This current study aims to evaluate the utility of Ab seroprevalence estimates as a surveillance tool for LF transmission. The objectives of the study were to (i) describe the seroprevalence of *Bm14* Ab*, Bm33* Ab, and *Wb123* Ab from the SaMELFS 2018 human survey, (ii) identify risk factors for Ab seropositivity, (iii) investigate age-specific associations between Ag and Ab, and (iv) evaluate any geographic clustering of seropositive participants.

## Methods

### Data source

The SaMELFS study design has been described elsewhere [14]. Briefly, a community-based sero-survey of participants ≥5 years old was conducted in 2018 in 35 PSUs consisting of either 1 or 2 villages. Thirty PSUs were randomly selected and 5 PSUs were purposively selected by the Ministry of Health as ‘suspected LF hotspots’ based on local knowledge and high prevalence from previous surveys [15]. In addition, a convenience survey of 5-9-year-old children was conducted in each PSU. Each participant completed a questionnaire to collect demographic information and participation in previous MDA programs and had a finger prick sample of up to 400μL of blood collected into heparin microtainers. Ag-positivity was detected using Alere™ Filariasis Test Strips (FTS) (Abbott, Scarborough, ME), and thick blood smears were prepared from Ag-positive samples to detect Mf.

### Multiplex bead assay

Dried blood spots (DBS) were eluted into 96-well plates and then diluted to a final concentration of 1:400 [16]. All samples were tested using MBA for antifilarial Ab to *Bm14, Bm33*, and *Wb123* [17]. Sample plates were read on a Bio-Plex 200 instrument (Bio-Rad, Hercules, CA). For internal quality control purposes, 4 controls were used for the MBA analyses: a buffer blank containing the assay buffer only (in order to subtract any background noise), 2 pools of reference sera from known Ab-positives, and finally a negative control serum with known negative LF status. For each antigen, the mean plus 3 standard deviations of the median fluorescence intensity-background (MFI-bg) of a panel of 81 individuals from non-endemic regions were used to determine a threshold for seropositivity.

### Definition of subgroups used in the analysis

Participants were considered (i) Ag-positive if they had a positive FTS result, (ii) Ab-positive if they were seropositive to at least one Ab, and (iii) LF-positive if they were seropositive to Ag and/or any Ab.

### Statistical analysis

Data were analyzed using Stata (StataCorp, Version 17.0, College Station, TX). Summary statistics with 95% confidence intervals (95% CI) for seroprevalence was calculated for age groups (5-9 years old vs ≥10 years old), gender (male or female), PSU selection (randomly selected vs purposively selected), and region (Apia Urban area [AUA], Northwest Upolu [NWU], Rest of Upolu [ROU], and Savai'i [SAV]). Where overall Ab and Ag prevalence were reported in all 35 PSUs, summary statistics were reported without 95% CIs.

Adjustment for selection probability and clustering has been described previously [14]. Briefly, weighting for selection probability and clustering were based on the 2016 Samoa Census [18] and performed using the ‘svyset’ command in Stata with PSU as the unit of clustering. Age group and gender standardized weights were applied using information from the 2016 Samoa Census [18]. Prevalence estimates for the 2 main age groups were adjusted for selection probability and clustering and standardized for gender but not age. The prevalence estimates for all ages ≥5 years were adjusted for selection probability and clustering and standardized for gender (except when comparing between genders) and age using 5-year age bands.

Prevalence ratios were used to compare the seroprevalence of poly-seropositivity with Ag and Ab by PSU selection and age group with differences tested using Fisher's Exact test. *P*-values were adjusted for multiple comparisons using the ‘qqvalue’ package in Stata. Associations between Ag and Ab prevalence at the PSU level in participants 5-9 years old and participants ≥5 years old were investigated using Spearman's correlation coefficient (Stata command ‘pwcorr’). Logistic regression was used to assess associations between demographic and behavioural data and LF positivity. Variables with *P*<0.2 on univariable analyses were tested using multivariable logistic regression; variables were sequentially removed from the multivariable models to arrive at the most parsimonious models, in which variables with *P*<0.05 were retained. Geographic clustering at the household, PSU, and regional levels were assessed using intra-cluster correlation coefficient (ICC) estimated using a multi-level logistic regression model (Stata commands ‘melogit’ and ‘estat icc’) adjusting for age and sex. Intra-cluster correlation assumes that observations in a cluster are more similar (homogenous) than observations outside a cluster, therefore observations within a cluster are correlated and those outside a cluster are independent [19]. ICC values range from 0 to 1 and measure how similar observations are; a higher ICC indicates that the outcome measure is more homogeneous (there is higher degree of clustering at that level).

## Results

### Sample inclusion and exclusion

Of 4420 participants recruited in 2018; 480 aged <5 years were excluded. The remaining 3940 participants aged ≥5 years had DBS collected for MBA, of whom 89 had no results available (reasons included low bead count, no consent to use samples for additional testing, and duplicate samples). A further 56 participants without an FTS result were excluded (reasons included invalid FTS results, insufficient blood), giving a total sample of 3795 participants included in the current study (please refer to Supplementary Figure 1).

### Study population demographics

The mean participant age was 20.7 years (range: 5-90 years); 51.2% were female and 40.9%, 19.2%, 22.9%, and 17.0% were from NWU, ROU, SAV, and AUA, respectively. Fourteen percent were recruited from purposively selected PSUs. Overall, 39.8% of participants aged 5-9 years were recruited via the convenience survey. There were no significant differences in demographic characteristics between purposively selected and randomly selected PSUs (Supplementary Table 1). However, significantly more participants from purposively selected PSUs had taken MDA in the past (80.7% vs 62.6%; *P*<0.001) and had spent their whole life in Samoa (95.0% vs 88.3%; *P*<0.001). No purposive PSUs were selected from AUA.

### Unadjusted seroprevalence of Ag and Ab

Overall, 117 (3.1%) participants were Ag-positive, 1889 (49.8%) seropositive for at least one Ab, and 1892 (49.9%) were LF-positive. The most prevalent Ab was *Bm33* Ab (43.7%), followed by *Wb123* Ab (26.0%), and *Bm14* Ab (15.4%). Of 3277 participants from randomly selected PSUs (1641 aged 5-9 years, 1636 aged ≥10 years), 1573 (48%) were LF-positive (37.1% [n=608] aged 5-9 years and 59.0% [n=965] aged ≥10 years). Of 518 participants from purposively selected PSUs (255 aged 5-9 years, 263 aged ≥10 years) 319 (61.6%) were LF-positive (47.1% [n=120] aged 5-9 years and 75.7% [n=199] aged ≥10 years).

### Adjusted seroprevalence by PSU selection, age, and sex

Adjusted Ag, *Bm14* Ab, *Wb123* Ab, and *Bm33* Ab prevalence in all 35 PSUs were 3.7%, 20.3%, 32.2%, and 51.0%, respectively. Significantly more participants from purposively selected PSUs were seropositive for Ag (*P*<0.001), *Bm14* Ab (*P*<0.001), *Bm33* Ab (*P=*0.02), and *Wb123* Ab (*P=*0.01) compared to randomly selected PSUs. Significantly higher prevalence of Ag and individual Abs was seen among participants aged ≥10 years compared to those aged 5-9 years. There were no significant differences in prevalence of Ag, Ab, and LF-positivity when 5-9-year-olds in purposively and randomly selected PSUs were compared. However, participants aged ≥10 years from purposively selected PSUs had significantly higher prevalence of Ag (*P*=0.001), *Bm14* Ab (*P=*0.003), *Bm33* Ab (*P=*0.05), and *Wb123* Ab (*P=*0.01) (Table 1 and Supplementary Figure 2).

**Table 1:** Antibody and antigen prevalence by age (adjusted for sampling design and standardised by sex) and PSU (adjusted for survey design and standardised by age and sex), Samoa 2018.

Total (N=3795)	Overall		Purposively selected PSUs									Randomly selected PSUs									*P-value 1*	*P-value 2*	*P-value 3*
	Total testing positive^		Total testing positive^			Age 5-9 years old testing positive*			Age ≥10 years old testing positive*			Total testing positive^			Age 5-9 years old testing positive*			Age ≥10 years old testing positive*					
	N	%	N	%	95% CI	N	%	95% CI	N	%	95% CI	N	%	95% CI	N	%	95% CI	N	%	95% CI			
Ag	117	3.7	31	10	(7.4-13.4)	4	2.1	(1.0-4.3)	27	11.4	(7.9-16.1)	86	3.5	(2.4-5.0)	24	1.3	(0.8-2.1)	62	4.1	(2.7-6.3)	**<0.001**	0.28	**0.001**
*Bm14 Ab*	583	20.3	125	36.4	(31.9-41.1)	24	9.6	(4.9-17.4)	101	37.8	(30.5-45.7)	458	19.8	(16.3-23.9)	123	6.8	(4.8-9.7)	335	22.2	(17.1-28.2)	**<0.001**	0.38	**0.003**
*Bm33 Ab*	1659	51.0	297	66.6	(57.1-74.8)	111	41.3	(30.3-53.3)	186	67.2	(56.8-76.2)	1362	50.5	(44.8-56.2)	509	30.3	(24.8-36.5)	853	54.3	(47.2-61.2)	**<0.001**	0.08	**0.048**
*Wb123 Ab*	987	32.2	184	47.9	(42.4-53.4)	49	18.6	(15.8-21.4)	135	49.7	(41.5-57.9)	803	31.7	(25.9-38.0)	265	15.6	(11.6-20.7)	538	34.4	(27.3-42.3)	**<0.001**	0.30	**0.012**
Any Ab	1889	57.8	317	70.7	(61.1-78.7)	119	44.4	(34.3-55.0)	198	72.0	(61.4-80.6)	1572	57.4	(51.2-63.4)	608	36.3	(30.5-42.6)	964	61.2	(53.5-68.4)	**<0.001**	0.18	0.11
Any Ag or Ab	1892	57.9	319	71.7	(61.3-80.2)	120	45.1	(34.9-55.8)	199	72.9	(61.8-81.6)	1573	57.4	(51.2-63.4)	608	36.4	(30.5-42.6)	965	61.3	(53.5-68.5)	**<0.001**	0.15	0.09

⁎ Adjusted for sampling design and standardised by sex.

^ Adjusted for survey design and standardised by age and sex. Ag: Antigen; Ab: Antibody; CI: Confidence Interval; PSU: Primary Sampling Unit. *P*-value 1: Testing for significant differences in prevalence between all study participants 5-9 years old and ≥10 years old; *P*-value 2: Testing for significant differences in prevalence between participants 5-9 years old in randomly and purposively selected PSUs; *P*-value 3: Testing for significant differences in prevalence between participants ≥10 years old in randomly and purposively selected PSUs.

Significantly higher prevalence of *Bm14* Ab (*P<*0.001) and *Wb123* Ab (*P<*0.001) were seen in males compared to females, but not for *Bm33* Ab, Ag or LF-positivity. Significantly more males from purposively selected PSUs were Ag (*P<*0.001), *Bm14* Ab (*P=*0.001), *Bm33* Ab (*P=*0.01), and *Wb123* Ab-positive (*P=*0.02) compared to males from randomly selected PSUs. Similarly, more females from purposively selected PSUs were positive for *Bm14* Ab (*P=*0.02), and *Wb123* Ab (*P=*0.05) compared to females from randomly selected PSUs (Table 2).

**Table 2:** Antibody, antigen, and microfilariae prevalence by sex (adjusted for sampling design and standardised by age), Samoa 2018.

	Total population				Purposively selected PSUs						Randomly selected PSUs						*P-value 1*	*P-value 2*	*P-value 3*
	Male		Female		Male			Female			Male			Female					
	N	%	N	%	N	%	95% CI	N	%	95% CI	N	%	95% CI	N	%	95% CI			
Ag	68	4.5	49	2.9	17	11.7	(10.3-13.3)	14	8.2	(4.5-14.5)	51	4.2	(3.0-5.8)	35	2.8	(1.4-5.3)	0.17	**<0.001**	0.06
*Bm14 Ab*	323	25.1	260	15.3	65	44.8	(42.5-47.0)	60	27.6	(18.9-38.3)	258	24.5	(19.8-29.9)	200	14.8	(11.7-18.6)	**<0.001**	**0.001**	**0.02**
*Bm33 Ab*	825	53.9	834	48.0	134	69.2	(62.8-74.9)	163	63.9	(51.3-74.8)	691	53.5	(47.1-59.7)	671	47.4	(40.5-54.4)	0.37	**0.01**	0.08
*Wb123 Ab*	545	37.5	442	26.5	91	55.4	(49.1-61.5)	93	40.1	(30.6-50.4)	454	37.0	(30.7-43.9)	349	25.9	(20.2-32.7)	**<0.001**	**0.02**	**0.045**
Any Abs	957	61.5	932	53.9	146	73.3	(68.3-77.8)	171	67.9	(53.6-79.5)	811	61.2	(54.9-67.2)	761	53.4	(46.1-60.6)	0.09	**0.02**	0.16
Any Ag or Ab	960	61.6	932	53.9	148	75.3	(68.7-81.0)	171	67.9	(53.6-79.5)	812	61.2	(54.9-67.2)	761	53.4	(46.1-60.6)	0.08	**0.01**	0.16

Ag: Antigen; Ab: Antibody; CI: Confidence Interval; PSU: Primary Sampling Unit.

*P*-value 1: Testing for significant differences between sex in all participants included in the study; *P*-value 2: Testing for significant differences between in prevalence between male participants in randomly and purposively selected PSUs; *P*-value 3: Testing for significant differences in prevalence between female participants in randomly and purposively selected PSUs.

**Figure 1:**
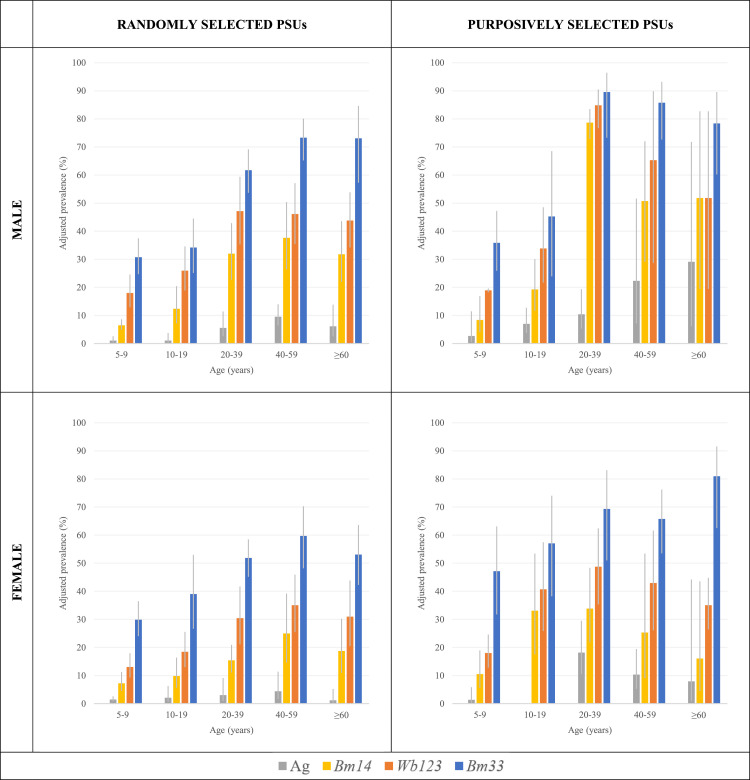
Prevalence of antigen and antibody among males from randomly (top left) and purposively selected PSUs (top right), and females from randomly (bottom left) and purposively selected PSUs (bottom right) adjusted for survey design, Samoa 2018.

The lowest prevalence of Ag and all 3 Ab were among participants aged 5-9 years irrespective of gender or PSU selection. For males and females from randomly selected PSUs, the highest prevalence of *Bm14* Ab (37.7% & 25.0%), *Bm33* Ab (73.3% & 59.7%), and Ag (9.6% & 4.4%) were seen among 40-59-year-olds, respectively. The highest prevalence of *Wb123* Ab was seen among 40-59-year-old females (35.0%) and 20-39-year-old males (47.2%). Among males from purposively selected PSUs, seroprevalence of *Bm33* Ab (89.6%)*, Wb123* Ab (84.8%)*,* and *Bm14* Ab (78.7%) was highest among males aged 20-39 years, though Ag prevalence (29.1%) was highest among ≥60-year-olds. Among females from purposive PSUs, *Wb123* Ab (48.8%) and Ag (18.2%) were highest among 20-39-year-olds, *Bm14* Ab (33.1%) among 10-19-year-olds, and *Bm33* Ab (80.9%) among ≥60-year-olds (Figure 1)

### Patterns of poly-seropositivity with Ag and Ab by age and PSU selection

**Figure 2:**
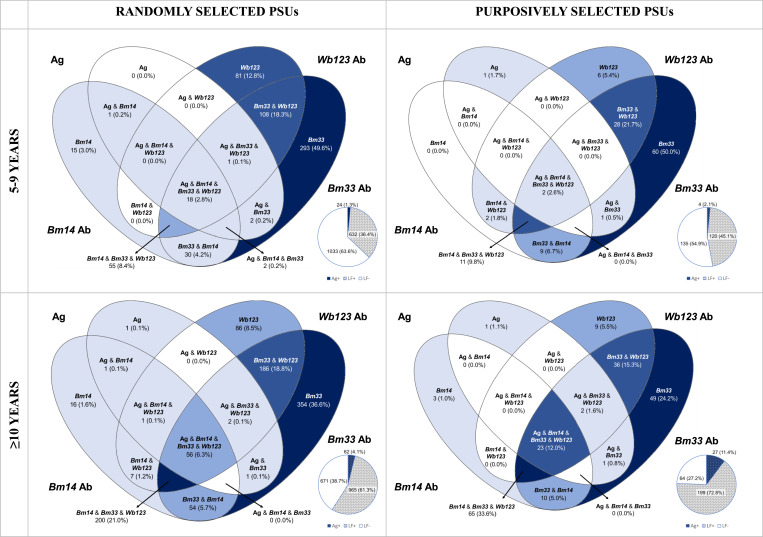
Venn diagrams to represent the prevalence among 1892 LF-positive participants by age and PSU selection, adjusted for survey design, Samoa 2018. Pie charts indicate the total individuals testing LF-positive (hashed slice) and LF-negative (white slice). The blue slice indicates the total LF-positive individuals who tested Ag-positive.

For all 1892 LF-positive participants, patterns of poly-seropositivity differed by age group (5-9 years vs ≥10 years) and PSU selection (randomly selected vs purposively selected). Overall, 0% of 5-9-year-olds from randomly selected PSUs tested positive to Ag only, 64.0% (n=389) to a single Ab, 23.0% (n=140) to 2 Ab, 0.5% (n=3) to Ag and one other Ab, 0.5% (n=3) to Ag and 2 Ab, 9.0% (n=55) to all 3 Ab, and 3.0% (n=18) to all seromarkers. For ≥10-year-olds from randomly selected PSUs, 0.1% (n=1) tested positive to Ag only, 47.3% (n=456) to a single Ab, 25.6% (n=247) to 2 Ab, 0.2% (n=2) to Ag and one other Ab, 0.3% (n=3) to Ag and 2 Ab, and 20.7 (n=200) to all 3 Ab, and 5.8% (n=56) to all seromarkers. Among 5-9-year-olds from purposively selected PSUs, 0.8% (n=1) tested positive to Ag only, 55.0% (n=66) to a single Ab, 32.5% (n=39) to 2 Ab, 0.8% (n=1) to Ag and one other Ab, 0% to Ag and 2 Ab, 9.2% (n=11) to all 3 Ab, and 1.7% (n=2) to all seromarkers. Lastly, 0.5% (n=1) of ≥10-year-olds from purposively selected PSUs tested positive to Ag only, 30.7% (n=61) to a single Ab, 23.1% (n=46) to 2 Ab, 0.5% (n=1) to Ag and one other Ab, 1.0% (n=2) to Ag and 2 Ab, 32.7% (n=65) to all 3 Ab, and 11.6% (n=23) to all seromarkers. Figure 2 presents a Venn diagram of poly-seropositivity of all LF-positive participants by age and PSU selection, adjusted for survey design.

Significantly more participants aged 5-9 years from randomly selected PSUs were seropositive for *Bm33* Ab only (*P*<0.001) and *Wb123* Ab only (*P*=0.04) compared to ≥10-year-olds in randomly selected PSUs. However, significantly fewer participants aged 5-9 years in randomly selected PSUs were seropositive for all 3 Abs only (*P*<0.001) and for all Abs and Ag (*P*=0.05) compared to ≥10-year-olds in randomly selected PSUs. In purposively selected PSUs, significantly more 5-9-year-olds were seropositive for *Bm33* Ab only (*P*<0.001), but fewer seropositives for all 3 Abs only (*P*<0.001) and all Abs and Ag (*P*=0.01). Significantly more 5-9-year-olds in randomly selected PSUs were seropositive for *Wb123* Ab only (*P*=0.04) compared to 5-9-year-olds in purposively selected PSUs. Significantly more participants aged ≥10 years in purposively selected PSUs were seropositive for all 3 Ab only (*P*=0.004) and all Ab and Ag (*P*=0.03), and fewer were seropositive for *Bm33* Ab only (*P*=0.01) when compared to those aged ≥10 years in randomly selected PSUs (Supplementary Table 2).

### Seroprevalence by region and randomly selected PSU

Seroprevalence to seromarkers did not differ significantly by region (Supplementary Figure 3 and Supplementary Table 3). The highest seroprevalence of *Bm14* Ab (23.6%), *Wb123* Ab (36.6%), and *Bm33* Ab (54.9%) were seen in participants from NWU. Among participants aged ≥10 years, 26.7%, 39.8%, and 59.0% of participants were seropositive to *Bm14* Ab, *Wb123* Ab, and *Bm33* Ab, respectively in NWU. The highest seroprevalence among 5-9-year-old participants was seen in ROU, where 10.3%, 20.2%, and 36.9% of participants were *Bm14* Ab, *Wb123* Ab, and *Bm33* Ab seropositive, respectively. The highest Ag prevalence among participants was found in AUA (4.4%), for participants aged 5-9 years in ROU (1.8%), and for participants aged ≥10 years in AUA (5.1%). Ag-positive participants were found in 28/35 PSUs, ranging from 1 to 13 positive participants per PSU. PSU-level Ag prevalence ranged from 0.2% to 20.0%. A higher proportion of participants aged ≥10 years were Ag-positive compared to those aged 5-9 years (range 0.0-18.7% vs 0.0-9.9%). Ab-positive participants were identified in all 35 PSUs. Participants aged 5-9 years were seropositive for *Bm14* Ab in 31/35 PSUs, for *Wb123* Ab in 34/35 PSUs, and for *Bm33* Ab in 35/35 PSUs (Supplementary Figure 4).

### Associations between seroprevalence in 5-9-year-olds and overall prevalence

**Figure 3:**
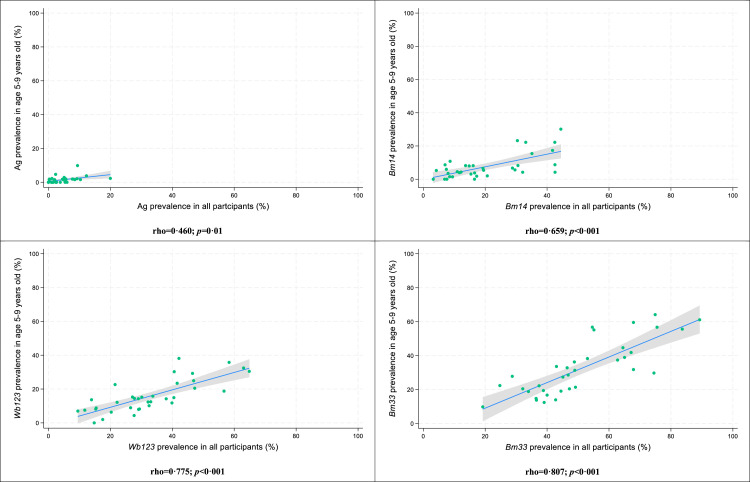
Correlation plots between antigen and antibody prevalence in participants aged 5-9 years and prevalence in all participants at the PSU level, Samoa 2018. Shaded areas represent 95% confidence intervals; green circles represent individual PSUs.

Significant associations but poor correlation was seen between prevalence at the PSU level among participants aged 5-9 years and participants ≥5 years (Figure 3). Correlation was strongest for *Bm33* Ab (Spearman's rho=0.807), followed by *Wb123* Ab (Spearman's rho=0.775), *Bm14* Ab (Spearman's rho=0.659), and Ag (Spearman's rho=0.460).

### Risk factor analysis

**Figure 4:**
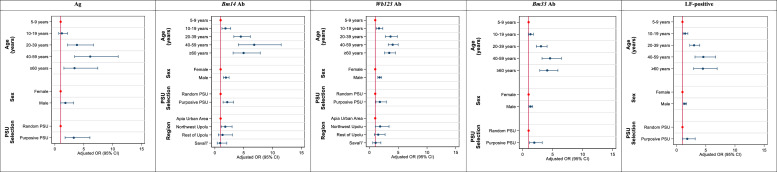
Summary of demographic and behavioural factors, and their associations with seropositivity for *Bm14* antibody, *Wb123* antibody, *Bm33* antibody, antigen and any antigen or antibody (LF-positive) using multivariable logistic regression, Samoa 2018. An odds ratio (OR) of 1.0 indicates no association between the exposure and outcome (seropositivity). An OR>1 indicates higher odds of association between the exposure and outcome. An OR<1 indicates lower odds of association between the exposure and outcome. The reference group is indicated by the red circles.

For all markers, male sex, older age groups, and residents of purposive PSUs were significantly more likely to be seropositive. The strongest association with male sex was seen for *Bm14* Ab, with 1.91 (95% CI: 1.48-2.47; *P*<0.001) the odds of testing positive compared to females. The strongest association with age was seen for *Bm14* Ab; 10-19-year-olds, 20-39-year-olds, 40-59-year-olds, and ≥60-year-olds had 1.84 (95% CI: 1.24-2.74; *P*=0.004), 4.50 (95% CI: 3.29-6.15; *P*<0.001), 6.83 (95% CI: 4.05-11.50; *P*<0.001), and 4.99 (95% CI: 3.16-7.88; *P*<0.001) the odds of seropositivity compared to 5-9-year-olds. For all seromarkers, 40-59-year-olds had the highest odds of seropositivity compared to 5-9-year-olds (*P*<0.001 for all markers). The strongest association with PSU selection was for Ag positivity; participants from purposively selected PSUs had 3.27 (95% CI: 1.76-6.07; *P*<0.001) the odds of testing Ag-positive compared to those from randomly selected PSUs. Residents of NWU were significantly more likely to test positive for *Bm14* Ab (aOR: 1.81, 95% CI: 1.10-2.99; *P*=0.02) and *Wb123* Ab (aOR: 1.85, 95% CI: 1.01-3.40; *P*=0.046) compared to other regions (Figure 4 and Supplementary Table 4).

### Intra-cluster correlation

Clustering of seropositive participants was significantly higher in households (ICC: Ag0.45 [95% CI: 0.30, 0.61], *Bm14* Ab0.32 [95% CI: 0.23, 0.42], *Bm33* Ab0.31 [95% CI: 0.24, 0.39], *Wb123* Ab0.29 [95% CI: 0.22, 0.38]) compared to PSUs or regions (Supplementary Figure 5). Clustering at the household level in purposively selected PSUs was higher than in randomly selected PSUs for *Wb123* Ab (ICC: 0.30 [95% CI: 0.16, 0.51]) and *Bm33* Ab (ICC: 0.35 [95% CI: 0.18, 0.56]), but was higher in randomly selected PSUs compared to purposively selected PSUs for Ag (ICC: 0.44 [95% CI: 0.27, 0.63]) and *Bm14* Ab (ICC: 0.29 [95% CI: 0.20, 0.40]) (Supplementary Figure 6).

## Discussion

Our study found that antifilarial Abs provided a more sensitive indicator of transmission than Ag and were useful for identifying high-risk populations and hotspots. Among all participants, 20.3%, 32.3%, and 51.0% tested seropositive to *Bm14* Ab, *Wb123* Ab, and *Bm33* Ab, respectively, whilst only 3.7% were Ag-positive. Poly-seropositivity for Ag and Ab was found to differ by age groups (aged 5-9 years and ≥10 years) and PSU selection (randomly selected and purposively selected PSUs). For all seromarkers, we found significant clustering at the household level compared to the PSU or regional level.

Our findings are similar to other reporting higher rates of Ab seroprevalence compared to Ag [5,20,21], suggesting that monitoring Ag alone may not accurately reflect transmission intensity, particularly in low prevalence settings. We also found significantly higher seroprevalence of Ag and Ab among participants aged ≥10 years compared to 5-9 years, and stronger correlation estimates of Ab prevalence in 5-9-year-olds with the overall population compared to Ag, suggesting Ab seroprevalence in 5-9-year-olds could be a good predictor of LF transmission at the community level. In American Samoa, Ab surveillance in school-based TAS found indications of ongoing LF transmission over a year earlier than Ag surveillance. Further, Ag prevalence estimates were seen to differ significantly between a school-based TAS of 6-7-year-olds and a community-based TAS of people aged ≥8 years, with estimates of 0.7% and 6.2%, respectively [21]. The implications of these findings are twofold; firstly, they suggest that measuring Ag prevalence alone in TAS may not be sufficiently sensitive for making programmatic decisions about stopping MDA or determining whether LF elimination targets have been reached. Secondly, they suggest that school-based TAS are not a good indicator of Ag prevalence in the community, or an accurate identifier of foci of ongoing transmission [1].

Such observations draw into question the appropriateness of monitoring LF prevalence among school-aged children to determine whether the critical threshold of <1% Ag prevalence has been reached. Whilst school-based TAS are more cost-effective and logistically efficient, they may not be accurately reflecting transmission in the community or be sufficiently sensitive to identify foci of ongoing LF transmission. Though our study suggests Ab prevalence in children aged 5-9 years may better correlate with prevalence in older age groups, overall Ab prevalence in still significantly lower in younger age groups, and there is a risk that residual pockets of ongoing transmission may be missed if prevalence is solely measured in younger age groups. As reported frequently [10,22], our study found significantly higher Ag and Ab seroprevalence among males compared to females. High seroprevalence in adult males suggest that they may be a reservoir of infection for LF, and future surveillance programmes should consider a targeted surveillance approach that includes older males to identify possible hotspots of infection [23]. It has been suggested that community-based Ab surveys of adults and older children may be more sensitive than school-based TAS of 6-7-year-old children for determining elimination thresholds [10]. Monitoring Ab seroprevalence may be a better measure of transmission for LF elimination programmes; however, further research is needed to better understand Ab kinetics and determine whether there are IgG thresholds that can be used to differentiate between active and past LF infection as well as targets for elimination.

In the 2018 SaMELFS study, the Samoan Ministry of Health purposively selected 5 PSUs as ‘suspected LF hotspots’. As has been previously reported [14], Ag prevalence was higher in these purposively selected PSUs compared to randomly selected PSUs. This study confirms similar Ab seroprevalence patterns, with significantly higher seropositivity to all 3 Ab in purposively selected PSUs. Our findings support those of Lau *et al.* [14], which emphasise the importance of incorporating local knowledge and co-design in the development of LF monitoring and surveillance strategies.

Our finding of intense clustering of positive seromarkers at the household level, supported by similar reports of higher ICC values for Mf, Ag, *Bm14* Ab, *Wb123* Ab, and *Bm33* Ab at the household level in American Samoa [3], suggest that LF infections and exposure are more intensely clustered within households. Our findings can further inform LF surveillance strategies wherein testing and treating household members (and possibly near neighbours) of any LF-positive individuals could be an effective means to eliminate pockets of residual LF transmission [3,14]. To date, treatment protocols have been based on Ag-positivity; however, to achieve the endgame of LF elimination, treatment of individuals and household members who are seropositive to Ag and Ab may be required. One important caveat to consider is the ambiguity around whether Ab-seropositivty is a marker or current LF infection or recent exposure, and the implications around treating individuals who may be ‘false-positives’. However, as MDA is given to whole populations irrespective of LF-positivity, such a method could be considered as a targeted MDA strategy for high-risk populations. This is the first report of LF Ab results from a large nationwide population representative serosurvey in Samoa, the results of which will act as a baseline of Ab seroprevalence to enable the understanding of Ab behaviour pre- and post-MDA, with Ab seroprevalence results 6-months following MDA soon to be available. An important limitation is that this study was conducted 7-11 weeks following MDA distribution, thus Mf prevalence would have been rapidly cleared from the population following treatment, preventing a comparison of Mf prevalence and Ab seroprevalence. However, we are confident that Ab seroprevalence reported reflects the pre-MDA epidemiology and can serve as a baseline for Ab prevalence in Samoa, given evidence of Ab persisting in the blood after treatment [24]. Further, adjustments were made to account for sampling design due to the inherent risk of selection bias when a cluster sampling methodology is adopted. Standardizing for participant age and sex accounted for the convenience survey of 5-9-year-olds. Whilst all efforts were made to mediate selection bias in the analysis of the study data and increase the accuracy of the results, there is still a risk that our results may have over- or underestimated the prevalence of seromarkers.

Our findings are particularly relevant as the *WHO Neglected Tropical Diseases Roadmap 2030* is rolled out globally, and as the WHO methodology to undertake TAS in IDA areas is being revised. Our results question whether using an Ag prevalence of <1% in 6-7-year-old children is an appropriate measure to determine whether a country has achieved elimination of LF, given that (i) significantly lower prevalence of both Ag and Ab among this demographic has been reported in several studies to date, and (ii) Ag prevalence is significantly lower than Ab seroprevalence rates, implying the lower sensitivity of Ag to detect LF exposure and infection. Further, whilst an individual can be infected with LF with only one bite, not all exposures will result in a viable worm infection, and it takes time for worm infections to establish. Therefore, young children are less likely to have detectable LF markers. Incorporating Ab surveillance into LF elimination programmes could be advantageous, given that Ab against filarial Ag can indicate infection prior to Ag or Mf detection [12]. Studies in Tanzania and Indonesia have used Ab prevalence over time as a means to measure the impact of MDA [25] and for stopping MDA decision-making [26].

A key limitation in LF surveillance is the absence of diagnostic tests that can identify active LF infections, and thus can signal sustained transmission. Rapid diagnostic tests for antifilarial Abs are in development; utilizing these tools in field surveys could provide more timely results to indicate active or recent LF transmission. However, there are concerns about low specificity of Abs due to persistent host responses to infection following clearance of the original infection. Our analysis of Abs 6-months post-MDA in Samoa will further elucidate Ab behaviour in Samoa.

## Conclusion

In conclusion, testing for Ab seroprevalence is a promising surveillance tool that can be adopted by countries aiming to eliminate LF. To fully understand how Ab surveillance can be used by LF programmes, further research is needed to establish a seroprevalence threshold to signal ongoing LF transmission and determine a threshold to indicate validation of elimination using Ab seroprevalence. Future cohort studies that monitor Ab levels of participants at baseline and following consecutive MDA rounds are required to elucidate the kinetics of Ab and Ag behaviour to determine whether monitoring Ab prevalence is an appropriate measure of LF transmission and infection.

## Declaration of competing interest

The authors declare no conflicts of interest.

## Appendix Supplementary materials

Image, application 1

Image, application 2

Image, application 3

Image, application 4

Image, application 5

Image, application 6

Image, application 7

Image, application 8

Image, application 9

Image, application 10
