# Supplementary material for: Anti-filarial antibodies are sensitive indicators of lymphatic filariasis transmission and enable identification of high-risk populations and hotspots
Source: Int J Infect Dis. 2024 Oct;147:None. doi: 10.1016/j.ijid.2024.107194 (PMC11530377; doi:10.1016/j.ijid.2024.107194)
Supplement: Supplementary file 1 [file mmc1.docx]

**Supplementary Table 1: Demographics of study population in randomly versus purposively selected PSUs, Samoa 2018.**

| **Variable** | **All participants**  **N=3795** | **Randomly selected PSU**  **N=3277** | **Purposively selected PSU**  **N=518** | ***P*-value** |
| --- | --- | --- | --- | --- |
| Age (years [SD]) | 20·7±19·1 | 20·8±19·2 | 20·0±18·6 | 0·39 |
| Age group (years) |  |  |  |  |
| 5-9 | 1896 (50·0) | 1641 (50·1) | 255 (49·2) | 0·72 |
| ≥10 | 1899 (50·0) | 1636 (49·9) | 263 (50·8) |  |
| Sex |  |  |  |  |
| Female | 1942 (51·2) | 1667 (50·9) | 275 (53·1) | 0·35 |
| Male | 1853 (48·8) | 1610 (49·1) | 243 (46·9) |  |
| Survey type |  |  |  |  |
| Household | 2283 (60·2) | 1970 (60·1) | 313 (60·4) | 0·89 |
| Convenience | 1512 (39·8) | 1307 (39·9) | 205 (39·6) |  |
| Household size (years) | 6·7±4·0 | 6·6±4·1 | 6·8±3·1 | 0·42 |
| Took MDA in 2018 |  |  |  |  |
| No | 349 (9·2) | 297 (9·1) | 52 (10·1) | 0·47 |
| Yes | 3441 (90·8) | 2976 (90·9) | 465 (89·9) |  |
| Taken MDA in the past |  |  |  |  |
| **No** | **1325 (35·0)** | **1225 (37·4)** | **100 (19·3)** | **<0·001** |
| **Yes** | **2466 (65·0)** | **2049 (62·6)** | **417 (80·7)** |  |
| Know about MDA |  |  |  |  |
| No | 280 (7·4) | 237 (7·2) | 43 (8·3) | 0·39 |
| Yes | 3515 (92·6) | 3040 (92·8) | 475 (91·7) |  |
| Time lived in Samoa |  |  |  |  |
| **Less than whole life** | **408 (10·8)** | **382 (11·7)** | **26 (5·0)** | **<0·001** |
| **Whole life** | **3387 (89·2)** | **2895 (88·3)** | **492 (95·0)** |  |
| Region |  |  |  |  |
| **AUA** | **644 (17·0)** | **644 (19·7)** | **0 (0·0)** | **<0·001** |
| **NWU** | **1552 (40·9)** | **1212 (37·0)** | **340 (65·6)** |  |
| **ROU** | **870 (22·9)** | **798 (24·4)** | **72 (13·9)** |  |
| **SAV** | **729 (19·2)** | **623 (19·0)** | **106 (20·5)** |  |

*Data are presented as number (%) or mean ± standard deviation.*
