# Supplementary material for: Anti-filarial antibodies are sensitive indicators of lymphatic filariasis transmission and enable identification of high-risk populations and hotspots
Source: Int J Infect Dis. 2024 Oct;147:None. doi: 10.1016/j.ijid.2024.107194 (PMC11530377; doi:10.1016/j.ijid.2024.107194)
Supplement: Supplementary file 2 [file mmc2.docx]

**Supplementary Table 2: Comparison of LF poly-seropositivity among 1892 LF-positive participants by sampling design and age groups, Samoa 2018**

| **Seropositivity** | **Randomly selected PSUs*** | | **Purposively selected PSUs*** | | **Ratio of proportion positive (*P*-value)^** | | | |
| --- | --- | --- | --- | --- | --- | --- | --- | --- |
|  | **5-9-years**  **N (%)** | **≥10 years**  **N (%)** | **5-9-years**  **N (%)** | **≥10 years**  **N (%)** | **5-9 years : ≥10 years  in random PSUs** | **5-9-years : ≥10 years in purposive PSUs** | **5-9-years in purposive : random PSUs** | **≥10 years in purposive : random PSUs** |
| **Total** | **608** | **965** | **120** | **199** |  |  |  |  |
| Ag only | 0 (0) | 1 (0.1) | 1 (1.6) | 1 (1.1) | 0.00 (1.000) | 1.45 (1.000) | 0.00 (0.442) | 11.0 (0.638) |
| *Bm14* Ab only | 15 (3.0) | 16 (1.6) | 0 (0) | 3 (1.0) | 1.88 (0.638) | 0.00 (0.638) | 0.00 (0.442) | 0.63 (1.000) |
| *Bm33* Ab only | 293 (49.5) | 354 (36.6) | 60 (50.0) | 49 (24.2) | 1.35 (**<0.001**) | 2.07 (**<0.001**) | 1.01 (1.000) | 0.66 (**0.007**) |
| *Wb123* Ab only | 81 (12.8) | 86 (8.5) | 6 (5.4) | 9 (5.5) | 1.51 (**0.040**) | 0.98 (1.000) | 0.42 (**0.043**) | 0.65 (0.193) |
| *Wb123* Ab and *Bm14* Ab only | 2 (0.2) | 7 (1.2) | 2 (1.8) | 0 (0.0) | 0.17 (0.872) | 0.00 (0.442) | 9.00 (0.442) | 0.00 (0.943) |
| *Wb123* Ab and *Bm33* Ab only | 108 (18.3) | 186 (18.8) | 28 (21.7) | 36 (15.3) | 0.97 (0.848) | 1.42 (0.638) | 1.19 (0.442) | 0.81 (1.000) |
| *Bm14* Ab and *Bm33* Ab only | 30 (4.2) | 54 (5.7) | 9 (6.7) | 10 (5.0) | 0.74 (0.968) | 1.34 (0.848) | 1.60 (0.638) | 0.88 (1.000) |
| Ag and *Bm14* Ab only | 1 (0.2) | 1 (0.1) | 0 (0) | 0 (0) | 2.00 (1.000) | NC | 0.00 (1.000) | 0.00 (1.000) |
| Ag and *Bm33* Ab only | 2 (0.2) | 1 (0.1) | 1 (0.5) | 1 (0.8) | 2.00 (0.898) | 0.63 (1.000) | 2.50 (0.820) | 8.00 (0.638) |
| Ag and *Wb123* Ab only | 0 (0) | 0 (0) | 0 (0) | 0 (0) | NC | NC | NC | NC |
| Ag and *Bm14* Ab and *Bm33* Ab only | 2 (0.2) | 0 (0) | 0 (0) | 0 (0) | 0.00 (0.442) | NC | 0.00 (1.000) | NC |
| Ag and *Bm14* Ab and *Wb123* Ab only | 0 (0.0) | 1 (0.1) | 0 (0) | 0 (0) | 0.00 (1.000) | NC | NC | 0.00 (1.000) |
| Ag and *Wb123* Ab and *Bm33* Ab only | 1 (0.1) | 2 (0.2) | 0 (0) | 2 (1.6) | 0.50 (1.000) | 0.00 (0.898) | 0.00 (1.000) | 8.00 (0.442) |
| *Bm14* Ab and *Wb123* Ab and *Bm33* Ab only | 55 (8.4) | 200 (21.0) | 11 (9.8) | 65 (33.6) | 0.40 (**<0.001**) | 0.29 (**<0.001**) | 1.17 (1.000) | 1.60 (**0.004**) |
| Ag and *Wb123* Ab and *Bm33* Ab and *Bm14* Ab | 18 (2.8) | 56 (6.3) | 2 (2.5) | 23 (12.0) | 0.44 (**0.046**) | 0.21 (**0.008)** | 0.89 (0.898) | 1.90 (**0.033**) |

**Adjusted for sampling design and standardised by age; ^Fisher’s Exact test for cell counts <20 adjusted for multiple comparisons; NC = not calculate*
