# Supplementary material for: Anti-filarial antibodies are sensitive indicators of lymphatic filariasis transmission and enable identification of high-risk populations and hotspots
Source: Int J Infect Dis. 2024 Oct;147:None. doi: 10.1016/j.ijid.2024.107194 (PMC11530377; doi:10.1016/j.ijid.2024.107194)
Supplement: Supplementary file 3 [file mmc3.docx]

**Supplementary Table 3: Antibody and antigen prevalence by region (adjusted for sampling design and standardised by age and sex) and age group (adjusted for survey design and standardised by sex), Samoa 2018**

|  |  |  | **Ag-positive** | ***Bm14* Ab** | ***Bm33* Ab** | ***Wb123* Ab** | **Any Abs** | **LF positive** |
| --- | --- | --- | --- | --- | --- | --- | --- | --- |
| **AUA** |  | **N** | 17 | 65 | 232 | 113 | 265 | 265 |
|  | **Overall** | **%** | 4.4 | 15.3 | 45.9 | 23.5 | 50.8 | 50.8 |
|  |  | **95% CI** | (2.2-8.5) | (11.9-19.5) | (37.1-54.9) | (19.6-27.8) | (42.2-59.4) | (42.2-59.4) |
|  |  | **N** | 2 | 11 | 75 | 24 | 85 | 85 |
|  | **5-9 years old** | **%** | 1.0 | 4.4 | 32.6 | 9.2 | 35.6 | 35.6 |
|  |  | **95% CI** | (0.4-2.5) | (2.6-7.5) | (17.5-52.3) | (5.4-15.3) | (20.7-54.0) | (20.7-54.0) |
|  |  | **N** | 15 | 54 | 157 | 89 | 180 | 180 |
|  | **≥10 years old** | **%** | 5.1 | 16.8 | 48.6 | 26.6 | 54.8 | 54.8 |
|  |  | **95% CI** | (2.5-10.1) | (13.9-20.3) | (39.9-57.5) | (21.7-32.2) | (46.0-63.3) | (46.0-63.3) |
| **NWU** |  | **N** | 41 | 221 | 576 | 367 | 659 | 659 |
|  | **Overall** | **%** | 3.8 | 23.6 | 54.9 | 36.6 | 62.7 | 62.7 |
|  |  | **95% CI** | (2.3-6.1) | (18.8-29.1) | (45.1-64.3) | (27.4-47.0) | (51.8-72.4) | (51.8-72.4) |
|  |  | **N** | 12 | 54 | 208 | 120 | 249 | 249 |
|  | **5-9 years old** | **%** | 1.3 | 6.6 | 28.8 | 16.7 | 35.4 | 35.4 |
|  |  | **95% CI** | (0.5-2.9) | (4.1-10.3) | (22.4-36.1) | (10.7-25.2) | (27.7-43.9) | (27.7-43.9) |
|  |  | **N** | 29 | 167 | 368 | 247 | 410 | 410 |
|  | **≥10 years old** | **%** | 4.6 | 26.7 | 59.0 | 39.8 | 67.0 | 67.0 |
|  |  | **95% CI** | (2.4-8.4) | (18.4-37.1) | (46.0-70.9) | (27.2-53.9) | (52.6-78.7) | (52.6-78.7) |
| **ROU** |  | **N** | 13 | 108 | 339 | 198 | 388 | 389 |
|  | **Overall** | **%** | 1.6 | 18.4 | 49.4 | 32.0 | 54.9 | 55.1 |
|  |  | **95% CI** | (0.8-3.5) | (10.7-29.6) | (41.4-57.5) | (24.3-40.8) | (47.8-61.9) | (48.0-61.9) |
|  |  | **N** | 6 | 40 | 144 | 78 | 171 | 171 |
|  | **5-9 years old** | **%** | 1.8 | 10.3 | 36.9 | 20.2 | 43.7 | 43.7 |
|  |  | **95% CI** | (0.8-3.7) | (4.6-21.6) | (25.5-50.1) | (11.5-33.2) | (32.1-56.1) | (32.1-56.1) |
|  |  | **N** | 7 | 68 | 195 | 120 | 217 | 218 |
|  | **≥10 years old** | **%** | 2.0 | 19.9 | 51.6 | 33.3 | 56.6 | 56.9 |
|  |  | **95% CI** | (0.8-4.6) | (10.4-34.6) | (43.0-60.2) | (23.6-44.7) | (49.2-63.8) | (49.6-63.9) |
| **SAV** |  | **N** | 15 | 64 | 215 | 125 | 260 | 260 |
|  | **Overall** | **%** | 2.7 | 13.8 | 42.7 | 25.1 | 50.0 | 50.0 |
|  |  | **95% CI** | (1.0-7.1) | (6.8-25.7) | (32.9-53.0) | (17.3-34.9) | (39.8-60.1) | (39.8-60.1) |
|  |  | **N** | 4 | 18 | 82 | 43 | 103 | 103 |
|  | **5-9 years old** | **%** | 1.1 | 6.2 | 22.8 | 12.1 | 29.2 | 29.2 |
|  |  | **95% CI** | (0.5-2.4) | (3.6-10.3) | (14.8-33.3) | (7.5-18.9) | (20.6-39.6) | (20.6-39.6) |
|  |  | **N** | 11 | 46 | 133 | 82 | 157 | 157 |
|  | **≥10 years old** | **%** | 3.6 | 15.8 | 47.4 | 27.5 | 54.9 | 54.9 |
|  |  | **95% CI** | (1.0-12.4) | (7.2-31.2) | (36.8-58.2) | (17.4-40.4) | (44.5-64.8) | (44.5-64.8) |
|  | | ***p-value 1*** | 0.441 | 0.242 | 0.129 | 0.333 | 0.197 | 0.197 |
|  |  | ***p-value 2*** | 0.776 | 0.258 | 0.469 | 0.196 | 0.499 | 0.499 |
|  |  | ***p-value 3*** | 0.499 | 0.233 | 0.284 | 0.186 | 0.189 | 0.191 |

*p-*value 1: Testing for significant differences between regions in all study participants in randomly selected PSUs; *p-*value 2: Testing for significant differences in prevalence between participants aged 5-9 years by region in randomly selected PSUs; *p-*value 3: Testing for significant differences in prevalence between participants ≥10 years by region in randomly selected PSUs. AUA (Apia Urban area) NWU (Northwest Upolu), ROU (Rest of Upolu), SAV (Savai’i).
