# Supplementary material for: Anti-filarial antibodies are sensitive indicators of lymphatic filariasis transmission and enable identification of high-risk populations and hotspots
Source: Int J Infect Dis. 2024 Oct;147:None. doi: 10.1016/j.ijid.2024.107194 (PMC11530377; doi:10.1016/j.ijid.2024.107194)
Supplement: Supplementary file 4 [file mmc4.docx]

# **Supplementary Table 4: Summary of demographic and behavioural factors, and their associations with seropositivity on univariable and multivariable logistic regression, Samoa 2018.**

|  |  | ***Bm14* Ab positive** | | | | | ***Bm33* Ab positive** | | | | | ***Wb123* Ab positive** | | | | | **LF-positive** | | | | | **Ag positive** | | | | |
| --- | --- | --- | --- | --- | --- | --- | --- | --- | --- | --- | --- | --- | --- | --- | --- | --- | --- | --- | --- | --- | --- | --- | --- | --- | --- | --- |
|  |  | **N (%)** | **OR** | **P-value** | **aOR** | **P-value** | **N (%)** | **OR** | **P-value** | **aOR** | **P-value** | **N (%)** | **OR** | **P-value** | **aOR** | **P-value** | **N (%)** | **OR** | **P-value** | **aOR** | **P-value** | **N (%)** | **OR** | **P-value** | **aOR** | **P-value** |
| **Total** | **3795 (100)** | **583 (15·4)** |  |  |  |  | **1659 (43·7)** |  |  |  |  | **987 (26·0)** |  |  |  |  | **1892 (49·9)** |  |  |  |  | **117 (3·1)** |  |  |  |  |
| **Age (years)** |  |  |  |  |  |  |  |  |  |  |  |  |  |  |  |  |  |  |  |  |  |  |  |  |  |  |
| 5-9 | 1896 (50·0) | 147 (25·2) | REF |  |  |  | 620 (37·4) |  |  |  |  | 314 (31·8) |  |  |  |  | 728 (38·5) | REF |  |  |  | 28 (23·9) | REF |  |  |  |
| 10-19 | 567 (14·9) | 72 (12·3) | 1·82 | 0·004 | **1·84** | **0·004** | 215 (13·0) | 1·33 | 0·073 | 1·32 | 0·092 | 135 (13·7) | 1·62 | 0·012 | **1·65** | **0·005** | 261 (13·8) | 1·47 | 0·007 | **1·46** | **0·009** | 10 (8·5) | 1·24 | 0·466 | 1·21 | 0·515 |
| 20-39 | 624 (16·4) | 158 (27·1) | 3·90 | <0·001 | **4·50** | **<0·001** | 365 (22·0) | 2·90 | <0·001 | **3·06** | **<0·001** | 246 (24·9) | 3·21 | <0·001 | **3·66** | **<0·001** | 398 (21·0) | 2·81 | <0·001 | **2·98** | **<0·001** | 31 (26·5) | 3·43 | <0·001 | **3·82** | **<0·001** |
| 40-59 | 481 (12·7) | 135 (23·2) | 6·00 | <0·001 | **6·83** | **<0·001** | 306 (18·4) | 4·40 | <0·001 | **4·57** | **<0·001** | 196 (19·9) | 3·61 | <0·001 | **4·03** | **<0·001** | 335 (17·7) | 4·39 | <0·001 | **4·58** | **<0·001** | 33 (28·2) | 5·72 | <0·001 | **6·11** | **<0·001** |
| ≥60 | 227 (6·0) | 71 (12·2) | 4·71 | <0·001 | **4·99** | **<0·001** | 153 (9·2) | 4·04 | <0·001 | **4·10** | **<0·001** | 96 (9·7) | 3·27 | <0·001 | **3·43** | **<0·001** | 170 (9·0) | 4·43 | <0·001 | **4·50** | **<0·001** | 15 (12·8) | 3·34 | 0·004 | **3·40** | **0·003** |
| **Sex** |  |  |  |  |  |  |  |  |  |  |  |  |  |  |  |  |  |  |  |  |  |  |  |  |  |  |
| Male | 1853 (48·8) | 323 (55·4) | 1·63 | <0·001 | **1·91** | **<0·001** | 825 (49·7) | 1·18 | 0·202 | **1·34** | **0·016** | 545 (55·2) | 1·51 | <0·001 | **1·75** | **<0·001** | 960 (50·7) | 1·24 | 0·067 | **1·39** | **0·002** | 68 (58·1) | 1·60 | 0·090 | **1·82** | **0·041** |
| Female | 1942 (51·2) | 260 (44·6) | REF |  |  |  | 834 (50·3) | REF |  |  |  | 442 (44·8) | REF |  |  |  | 932 (49·3) |  |  |  |  | 49 (41·9) | REF |  |  |  |
| **Sampling** |  |  |  |  |  |  |  |  |  |  |  |  |  |  |  |  |  |  |  |  |  |  |  |  |  |  |
| Random | 3277 (86·4) | 458 (78·6) | REF |  |  |  | 1362 (82·1) | REF |  |  |  | 803 (81·4) | REF |  |  |  | 1573 (83·1) | REF |  |  |  | 86 (73·5) | REF |  |  |  |
| Purposive | 518 (13·6) | 125 (21·4) | 1·96 | 0·003 | **2·15** | **0·001** | 297 (17·9) | 1·66 | 0·056 | **1·95** | **0·013** | 184 (18·6) | 1·71 | 0·019 | **1·81** | **0·018** | 319 (16·9) | 1·57 | 0·111 | **1·82** | **0·036** | 31 (26·5) | 2·79 | 0·002 | **3·27** | **<0·001** |
| **Region** |  |  |  |  |  |  |  |  |  |  |  |  |  |  |  |  |  |  |  |  |  |  |  |  |  |  |
| AUA | 644 (17·0) | 65 (11·1) | REF |  |  |  | 232 (14·0) | REF |  |  |  | 113 (11·4) | REF |  |  |  | 265 (14·0) | REF |  |  |  | 17 (14·5) | REF |  |  |  |
| NWU | 1552 (40·9) | 303 (52·0) | 1·72 | 0·035 | **1·81** | **0·022** | 781 (47·1) | 1·40 | 0·281 |  |  | 485 (49·1) | 1·76 | 0·065 | **1·85** | **0·046** | 880 (46·5) | 1·52 | 0·205 |  |  | 67 (57·3) | 0·91 | 0·843 |  |  |
| ROU | 870 (22·9) | 135 (23·2) | 1·23 | 0·604 | 1·34 | 0·479 | 395 (23·8) | 1·10 | 0·751 |  |  | 236 (23·9) | 1·40 | 0·247 | 1·52 | 0·153 | 446 (23·6) | 1·09 | 0·756 |  |  | 15 (12·8) | 0·39 | 0·088 |  |  |
| SAV | 729 (19·2) | 80 (13·7) | 0·93 | 0·843 | 0·93 | 0·850 | 251 (15·1) | 0·84 | 0·534 |  |  | 153 (15·5) | 1·03 | 0·932 | 1·06 | 0·861 | 301 (15·9) | 0·89 | 0·673 |  |  | 18 (15·4) | 0·67 | 0·544 |  |  |
| **Aware of MDA** | |  |  |  |  |  |  |  |  |  |  |  |  |  |  |  |  |  |  |  |  |  |  |  |  |  |
| No | 280 (7·4) | 61 (10·5) | REF |  |  |  | 140 (8·4) | REF |  |  |  | 94 (9·5) | REF |  |  |  | 159 (8·4) | REF |  |  |  | 10 (8·5) | REF |  |  |  |
| Yes | 3515 (92·6) | 522 (89·5) | 0·81 | 0·578 |  |  | 1519 (91·6) | 0·88 | 0·541 |  |  | 893 (90·5) | 0·88 | 0·651 |  |  | 1733 (91·6) | 0·88 | 0·529 |  |  | 107 (91·5) | 0·92 | 0·813 |  |  |
| **Time lived in Samoa** | |  |  |  |  |  |  |  |  |  |  |  |  |  |  |  |  |  |  |  |  |  |  |  |  |  |
| Other | 408 (10·8) | 63 (10·8) | REF |  |  |  | 154 (9·3) | REF |  |  |  | 94 (9·5) | REF |  |  |  | 182 (9·6) | REF |  |  |  | 11 (9·4) | REF |  |  |  |
| Whole life | 3387 (89·2) | 520 (89·2) | 0·99 | 0·972 |  |  | 1505 (90·7) | 1·41 | 0·141 |  |  | 893 (90·5) | 1·31 | 0·155 |  |  | 1710 (90·4) | 1·46 | 0·071 |  |  | 106 (90·6) | 1·06 | 0·859 |  |  |
| **Household size** | |  |  |  |  |  |  |  |  |  |  |  |  |  |  |  |  |  |  |  |  |  |  |  |  |  |
|  | 6·8 [4·1] | 6·6 [3·9] | 1·00 | 0·838 |  |  | 6·5 [3·9] | 0·98 | 0·053 |  |  | 6·7 [4·0] | 0·99 | 0·679 |  |  | 6·6 [4·0] | 0·98 | 0·142 |  |  | 6·4 [4·4] | 1·02 | 0·555 |  |  |

*Regions: AUA (Apia Urban area) NWU (Northwest Upolu), ROU (Rest of Upolu), SAV (Savai’i); CI: Confidence Interval; aOR: Adjusted Odds Ratio; MDA: Mass Drug Administration*
