# Supplementary figures and images for: Anti-filarial antibodies are sensitive indicators of lymphatic filariasis transmission and enable identification of high-risk populations and hotspots
Source: Int J Infect Dis. 2024 Oct;147:None. doi: 10.1016/j.ijid.2024.107194 (PMC11530377; doi:10.1016/j.ijid.2024.107194)

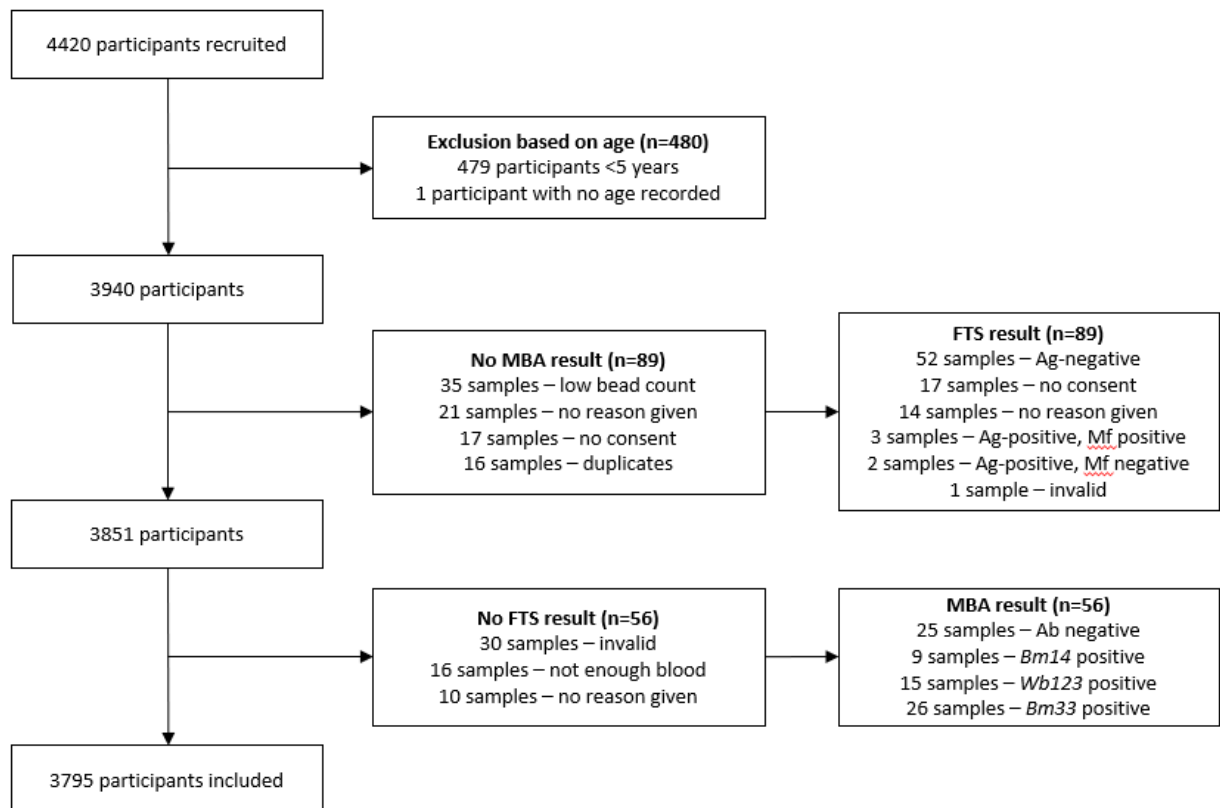

**Supplementary Figure 1: Flow chart of participant inclusion and exclusion, Samoa 2018**

Supplement: Supplementary file 5 [file mmc5.pdf]
