# Supplementary material for: Anti-filarial antibodies are sensitive indicators of lymphatic filariasis transmission and enable identification of high-risk populations and hotspots
Source: Int J Infect Dis. 2024 Oct;147:None. doi: 10.1016/j.ijid.2024.107194 (PMC11530377; doi:10.1016/j.ijid.2024.107194)
Supplement: Supplementary file 6 [file mmc6.pdf]

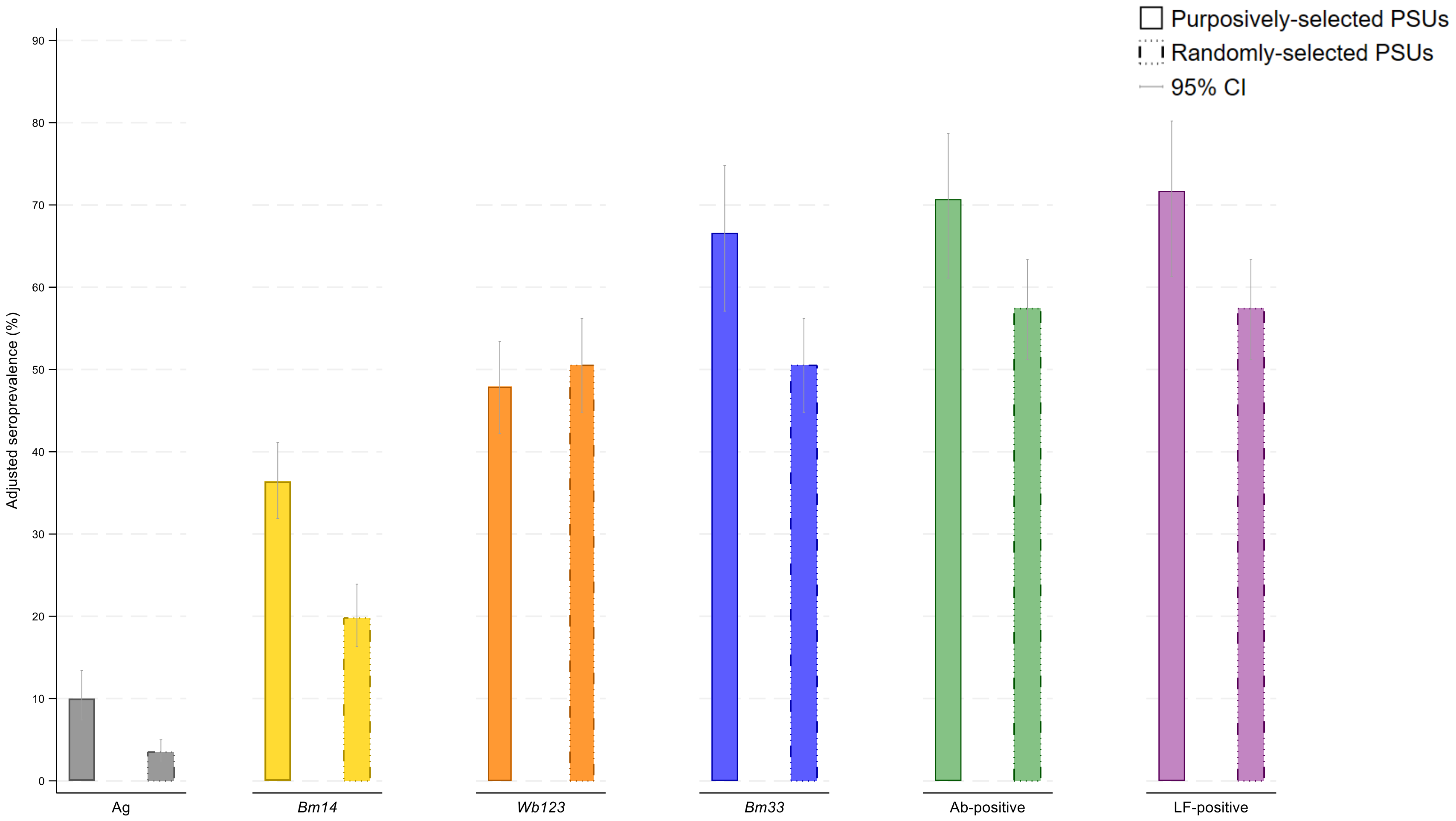

Supplementary Figure 2: Antibody and antigen prevalence by all PSUs (adjusted for survey design) and purposively- or randomly selected PSUs (adjusted for survey design and standardised by age and sex), Samoa 2018. Ab-positive indicates individuals testing seropositivity for at least one antibody. LF-positive indicates individuals testing seropositive for at least one LF marker (antibody and/or antigen)
