# Supplementary material for: Anti-filarial antibodies are sensitive indicators of lymphatic filariasis transmission and enable identification of high-risk populations and hotspots
Source: Int J Infect Dis. 2024 Oct;147:None. doi: 10.1016/j.ijid.2024.107194 (PMC11530377; doi:10.1016/j.ijid.2024.107194)
Supplement: Supplementary file 7 [file mmc7.pdf]

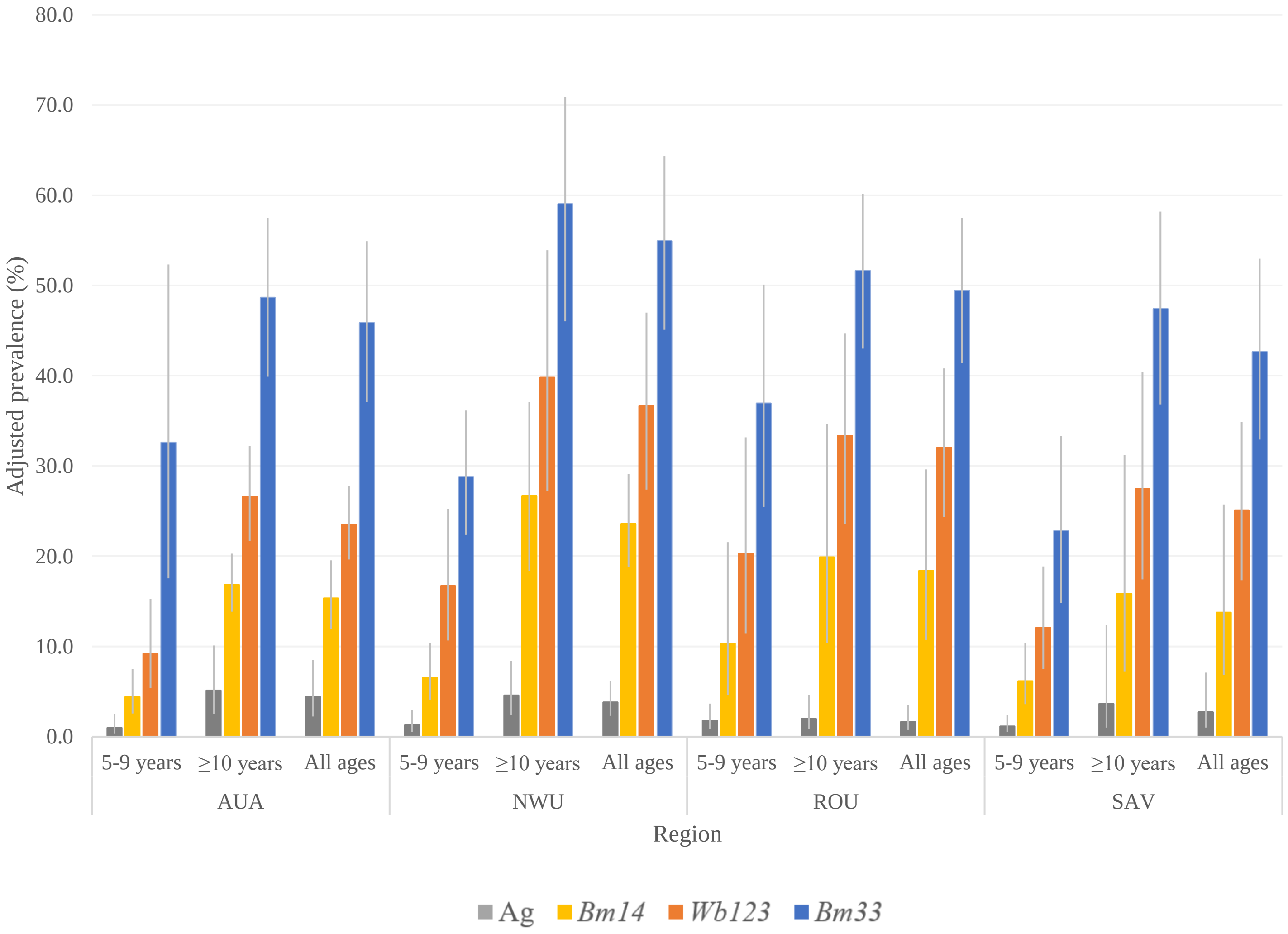

Supplementary Figure 3: Adjusted Ag and Ab prevalence by region for participants aged ≥5 years, 5-9 years, and ≥10 years from randomly selected PSUs, Samoa 2018. AUA (Apia Urban area) NWU (Northwest Upolu), ROU (Rest of Upolu), SAV (Savai'i)
