# Supplementary material for: Anti-filarial antibodies are sensitive indicators of lymphatic filariasis transmission and enable identification of high-risk populations and hotspots
Source: Int J Infect Dis. 2024 Oct;147:None. doi: 10.1016/j.ijid.2024.107194 (PMC11530377; doi:10.1016/j.ijid.2024.107194)
Supplement: Supplementary file 8 [file mmc8.pdf]

	5-9 years					>10 years					All participants				
	Ag	*Bm14*	*Wb123*	*Bm33*	LF positive	Ag	*Bm14*	*Wb123*	*Bm33*	LF positive	Ag	*Bm14*	*Wb123*	*Bm33*	LF positive
AUA	0.0	4.5	2.0	16.7	23.2	0	14.7	20.5	41.0	46.6	0.0	11.0	17.5	40.0	45.6
	0.0	0.0	7.5	18.8	20.7	5.2	15.7	20.0	42.9	47.2	2.4	7.6	11.7	34.0	36.2
	0.0	4.3	4.4	32.8	35.0	0.0	7.3	27.9	50.2	55.9	0.0	12.2	27.7	46.5	50.5
	2.0	8.2	8.2	20.4	20.4	8.1	15.7	20.2	37.2	41.3	5.6	13.7	15.3	32.2	34.8
	1.9	6.6	14.5	55.2	57.5	8.8	20.1	31.4	58.6	64.4	7.7	19.3	27.8	55.1	60.1
	0.0	0.0	10.2	13.8	20.2	5.5	22.7	36.7	43.9	56.5	5.4	16.6	32.5	36.6	46.5
NWU	1.6	3.1	12.5	31.8	34.9	4.1	22.4	34.1	71.9	77.9	2.0	15.4	33.1	67.9	73.7
	0.0	8.0	8.0	14.0	21.9	2.1	21.1	36.8	40.1	49.0	1.3	14.8	29.1	42.8	50.0
	0.0	2.0	12.4	21.4	32.4	1.7	24.3	37.2	56.2	62.9	1.5	20.7	32.2	49.2	57.6
	0.0	8.2	15.8	27.4	35.5	2.2	14.3	34.0	48.6	57.6	0.9	16.1	33.7	45.1	54.0
	0.0	1.4	7.0	22.2	23.7	0.0	10.4	12.9	40.0	49.3	0.0	9.5	9.5	37.4	45.6
	1.9	30.1	35.8	64.1	67.9	12.4	56.7	65.3	80.5	91.0	7.9	44.5	58.4	74.9	84.9
	1.6	8.3	11.8	38.9	45.6	9.0	36.1	45.3	72.6	77.5	8.5	30.6	39.9	65.0	70.8
	0.0	3.8	15.3	19.1	26.6	0.0	21.1	30.6	49.8	51.5	0.0	16.6	27.1	44.5	46.4
	1.7	5.4	23.4	38.3	45.2	6.2	25.9	48.8	55.0	58.7	5.7	19.5	41.6	53.0	56.3
	9.9	17.3	32.5	55.6	58.0	10.6	46.0	64.6	86.9	90.6	9.4	41.8	63.0	83.7	86.7
	1.8	6.7	29.3	29.7	42.5	8.7	40.8	58.5	78.2	89.5	4.5	28.8	46.5	74.5	83.1
	2.1	4.2	14.2	44.7	49.0	9.0	37.8	42.0	72.9	79.4	9.3	32.1	38.0	64.5	71.2
	3.8	15.5	20.5	41.9	45.7	10.8	35.4	46.6	68.2	75.3	12.3	35.1	47.2	67.1	75.0
	2.3	4.2	18.8	56.7	58.6	18.7	50.3	64.1	77.8	81.3	20.0	42.6	56.7	75.5	78.1
ROU	0.0	1.8	14.3	28.6	39.2	0.0	20.0	30.4	48.6	55.1	0.0	17.3	29.0	46.8	53.7
	0.0	1.5	6.3	14.8	19.3	3.9	7.8	16.9	37.6	45.5	2.6	8.5	20.3	36.5	43.1
	1.9	6.0	8.2	31.3	35.5	0.0	7.9	31.5	49.2	57.0	0.3	7.5	29.4	49.0	56.8
	0.0	8.7	13.7	22.4	29.3	0.0	5.4	15.6	28.9	33.8	0.0	7.0	13.9	24.8	28.1
	1.6	0.0	22.7	33.6	49.2	0.0	10.2	22.3	51.4	58.3	0.2	6.9	21.5	43.0	53.0
	4.7	23.2	38.1	56.8	61.9	3.3	36.0	47.2	60.2	61.8	2.3	30.4	42.2	54.6	56.7
	2.9	22.2	30.3	59.6	62.4	6.6	34.7	47.2	69.6	73.7	5.1	33.1	40.6	67.9	71.9
	0.0	22.2	30.5	61.1	61.1	6.9	55.1	74.7	95.4	97.7	3.8	42.5	64.9	89.3	92.0
SAV	0.0	0.0	0.0	9.8	9.8	2.3	6.1	19.8	21.7	31.6	1.2	3.2	14.8	19.2	27.1
	1.8	3.5	12.2	19.4	28.0	0.0	13.5	29.4	48.3	56.9	0.3	8.0	22.2	38.8	46.2
	0.0	10.8	8.8	12.4	20.0	0.0	9.5	11.8	49.7	49.7	0.0	8.7	15.4	39.1	40.4
	2.2	5.3	9.0	27.8	34.5	1.8	5.3	28.6	41.1	55.3	1.1	4.2	26.5	28.8	49.9
	1.5	3.9	15.2	36.3	42.4	4.1	18.9	32.7	52.0	58.6	4.8	11.7	30.2	48.8	59.0
	1.6	8.7	24.9	37.4	43.9	17.1	49.9	55.0	70.3	79.2	10.3	42.5	46.9	62.8	69.5
	0.0	5.6	14.9	20.4	26.0	6.7	27.4	41.4	49.2	53.7	6.0	29.6	40.5	47.2	50.8

Adjusted prevalence

0.0
20.0
40.0
60.0
80.0
100.0

Supplementary Figure 4: Adjusted antigen and antibody prevalence by PSU and region for participants aged 5-9 years, ≥10 years, and all study participants, Samoa 2018. Purposively selected PSUs are indicated by the purple borders. Colour spectrum from light to dark indicates progressive increase in prevalence. Light green denotes low prevalence, blue denotes high prevalence. AUA (Apia Urban area) NWU (Northwest Upolu), ROU (Rest of Upolu), SAV (Savai'i)
