# Supplementary material for: Anti-filarial antibodies are sensitive indicators of lymphatic filariasis transmission and enable identification of high-risk populations and hotspots
Source: Int J Infect Dis. 2024 Oct;147:None. doi: 10.1016/j.ijid.2024.107194 (PMC11530377; doi:10.1016/j.ijid.2024.107194)
Supplement: Supplementary file 9 [file mmc9.pdf]

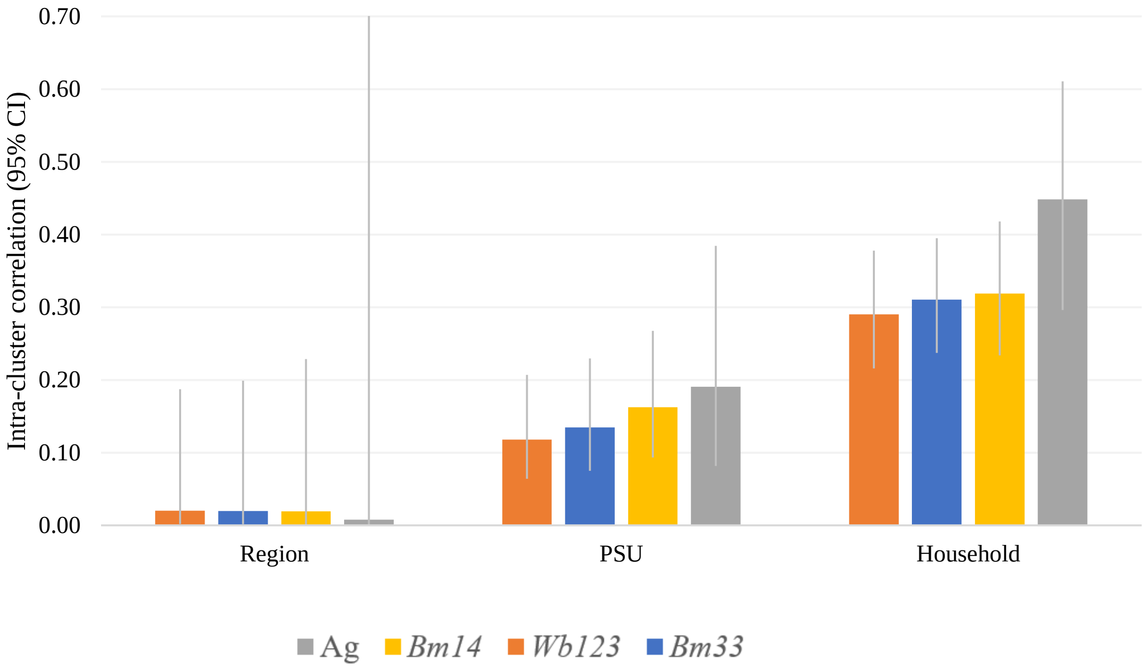

Supplementary Figure 5: Intra-cluster correlation and 95% CI at PSU and household levels for positive results for Ag, *Bm14* Ab, *Wb123* Ab, and *Bm33* Ab, Samoa 2018.
