# Supplementary material for: Anti-filarial antibodies are sensitive indicators of lymphatic filariasis transmission and enable identification of high-risk populations and hotspots
Source: Int J Infect Dis. 2024 Oct;147:None. doi: 10.1016/j.ijid.2024.107194 (PMC11530377; doi:10.1016/j.ijid.2024.107194)
Supplement: Supplementary file 10 [file mmc10.pdf]

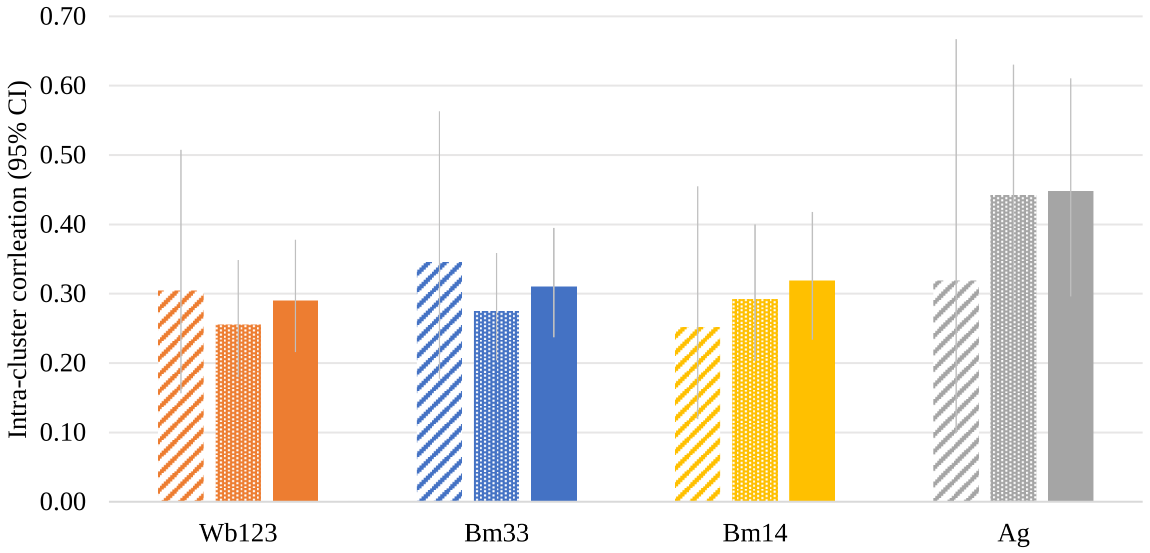


**Supplementary Figure 7: Intra-cluster correlation and 95% CI at household levels for positive results for antigen, *Bm14* antibody, *Wb123* antibody, and *Bm33* antibody.**

Antibody in all PSUs (solid bars), purposively selected PSUs (striped bars), and randomly selected PSUs (spotted bars), Samoa 2018**.**
